# Supplementary material for: Oral SARS-CoV-2 host responses predict the early COVID-19 disease course
Source: Sci Rep. 2024 Sep 18;14:21788. doi: 10.1038/s41598-024-67504-w (PMC11411107; doi:10.1038/s41598-024-67504-w)
Supplement: Supplementary file 1 — Supplementary Information 1. [file 41598_2024_67504_MOESM1_ESM.docx]

UNC OBSc Working Group

UNC OBSc Working Group representative

Natalie Bowman

nbowman@med.unc.edu

P: 984-974-7198

F: 984-974-4587

111 Mason Farm Rd

Medical Biomolecular Research Building, Room 2341b

Chapel Hill, NC 27599

Natalie Bowman [nbowman@med.unc.edu](mailto:nbowman@med.unc.edu) UNC School of Medicine, University of North Carolina, Chapel Hill, NC

David Wohl [david_wohl@med.unc.edu](mailto:david_wohl@med.unc.edu), UNC School of Medicine, Division of Infectious Diseases, Institute for Global Health and Infectious Diseases, University of North Carolina at Chapel Hill

Matt Wolfgang [matthew_wolfgang@med.unc.edu](mailto:matthew_wolfgang@med.unc.edu), Microbiology and Immunology, University of North Carolina at Chapel Hill

Alena Markmann [Alena.Markmann@unchealth.unc.edu](mailto:Alena.Markmann@unchealth.unc.edu) UNC School of Medicine, Division of Infectious Diseases, Institute for Global Health and Infectious Diseases, University of North Carolina at Chapel Hill

Erin Hoffman [erin_hoffman@med.unc.edu](mailto:erin_hoffman@med.unc.edu), Institute for Global Health and Infectious Diseases, University of North Carolina at Chapel Hill

Catherine Kronk, [catherine_kronk@med.unc.edu](mailto:catherine_kronk@med.unc.edu), Institute for Global Health and Infectious Diseases, University of North Carolina at Chapel Hill

Olivia Mitchem, [oliviami@email.unc.edu](mailto:oliviami@email.unc.edu), Lineberger Comprehensive Cancer Center, University of North Carolina at Chapel Hill

Camille O'Reilly [camille_oreilly@med.unc.edu](mailto:camille_oreilly@med.unc.edu), UNC School of Medicine, University of North Carolina at Chapel Hill

Aravinda de Silva [aravinda_desilva@med.unc.edu](mailto:aravinda_desilva@med.unc.edu), Microbiology and Immunology, University of North Carolina at Chapel Hill

Will Lovell [wlovell@unc.edu](mailto:wlovell@unc.edu), Microbiology and Immunology, University of North Carolina at Chapel Hill

S.T. Phillips [STP@email.unc.edu](mailto:STP@email.unc.edu), Adams School of Dentistry, University of North Carolina at Chapel Hill

Kathy Ramsey [caryramsey@gmail.com](mailto:caryramsey@gmail.com), Adams School of Dentistry, University of North Carolina at Chapel Hill

Jo-Ann Blake, [Jo-Ann_Blake@unc.edu](mailto:Jo-Ann_Blake@unc.edu), Adams School of Dentistry, University of North Carolina at Chapel Hill

Rob Maile, [robert_maile@med.unc.edu](mailto:robert_maile@med.unc.edu), Dept. of Surgery, University of North Carolina at Chapel Hill
