# Supplementary figures and images for: Oral SARS-CoV-2 host responses predict the early COVID-19 disease course
Source: Sci Rep. 2024 Sep 18;14:21788. doi: 10.1038/s41598-024-67504-w (PMC11411107; doi:10.1038/s41598-024-67504-w)

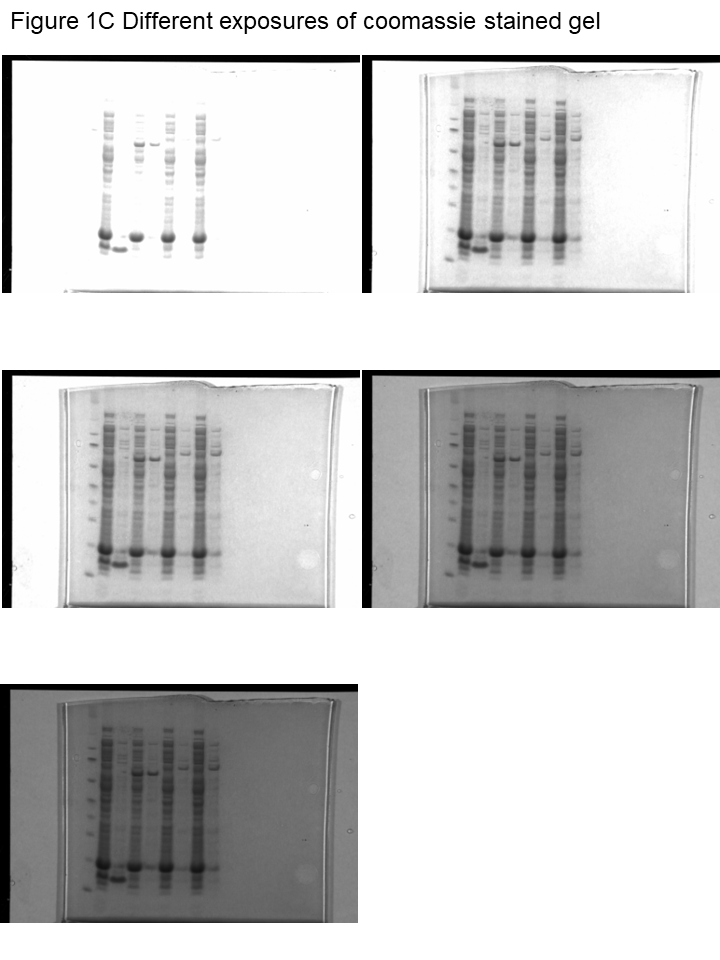

Supplement: Supplementary file 4 — Supplementary Information 4. [file 41598_2024_67504_MOESM4_ESM.tif]

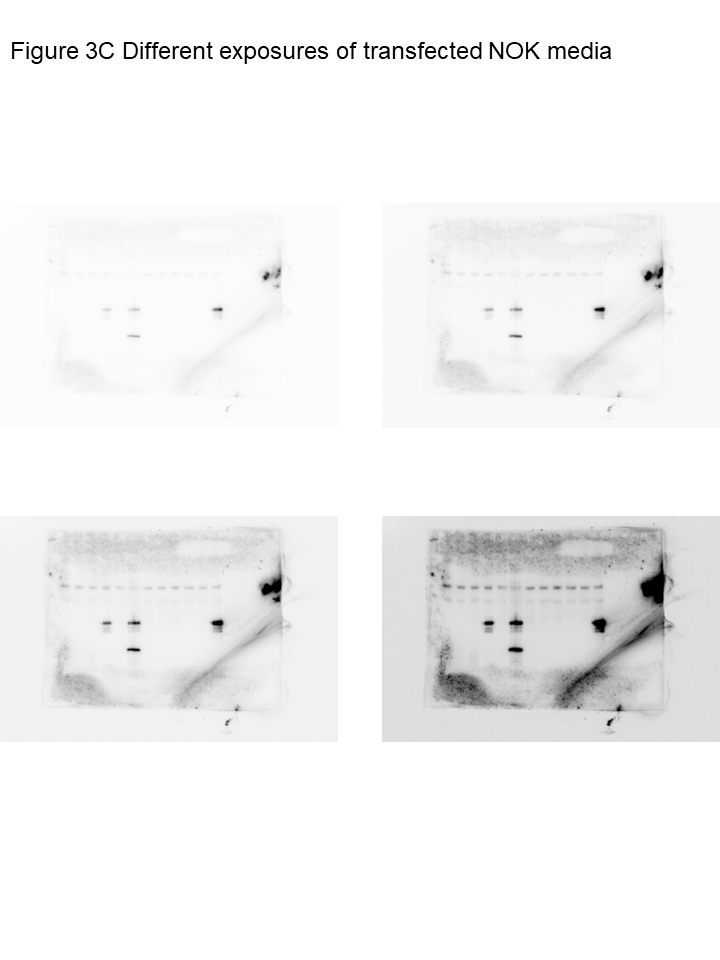

Supplement: Supplementary file 5 — Supplementary Information 5. [file 41598_2024_67504_MOESM5_ESM.tif]

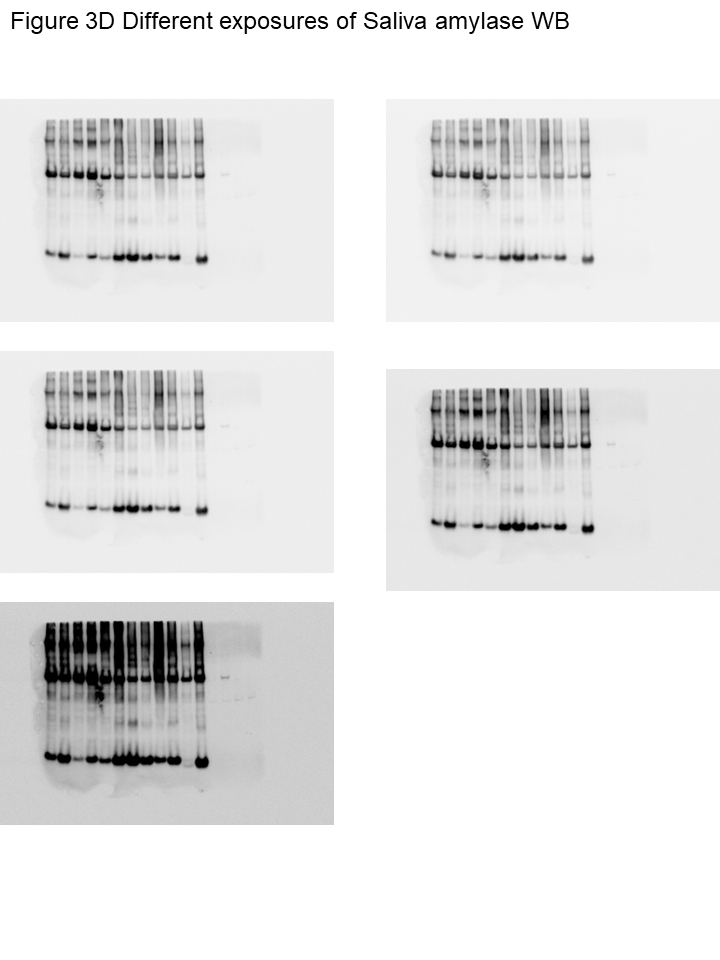

Supplement: Supplementary file 6 — Supplementary Information 6. [file 41598_2024_67504_MOESM6_ESM.tif]

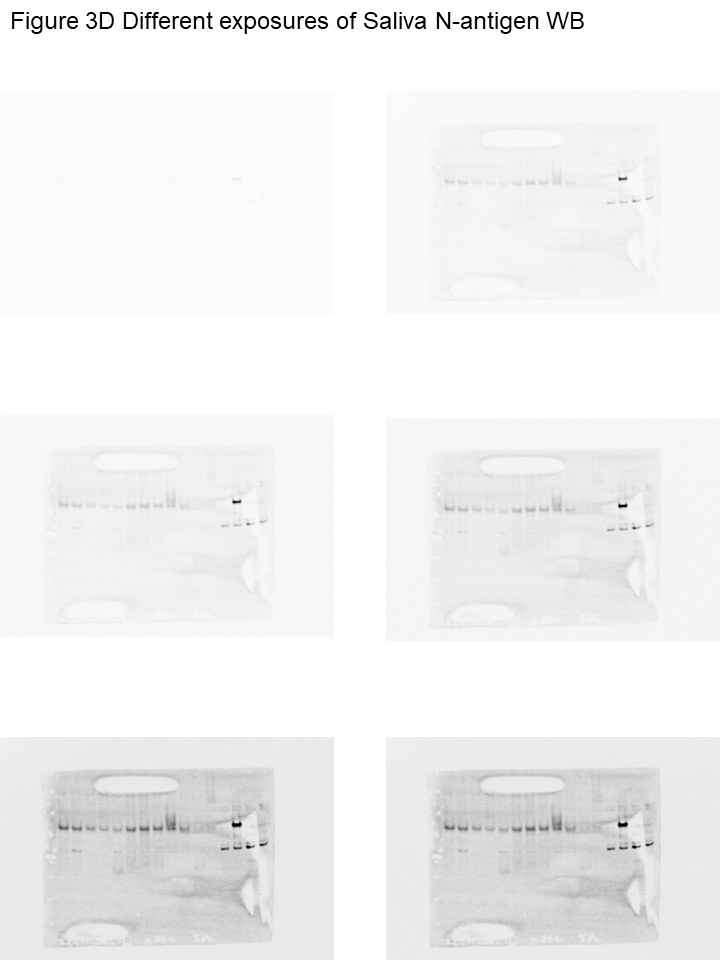

Supplement: Supplementary file 7 — Supplementary Information 7. [file 41598_2024_67504_MOESM7_ESM.tif]

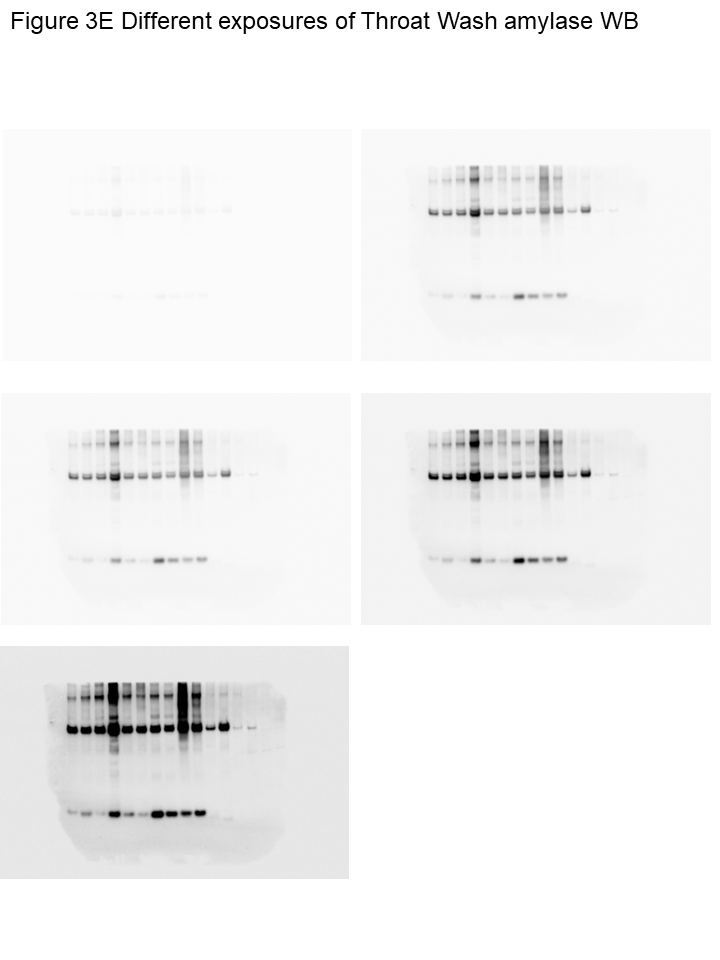

Supplement: Supplementary file 8 — Supplementary Information 8. [file 41598_2024_67504_MOESM8_ESM.tif]

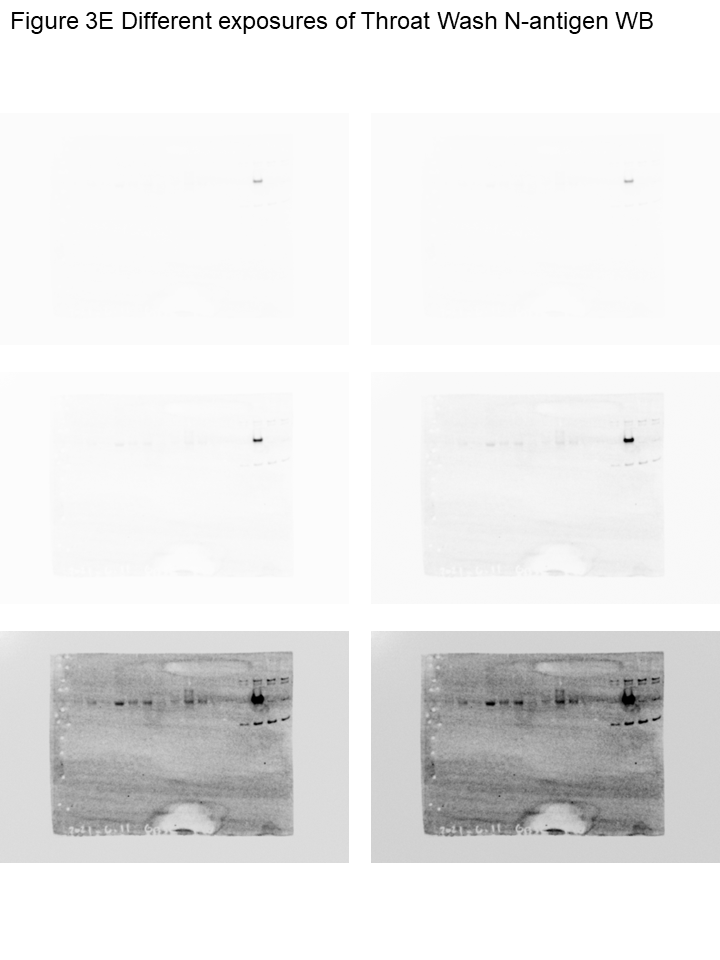

Supplement: Supplementary file 9 — Supplementary Information 9. [file 41598_2024_67504_MOESM9_ESM.tif]

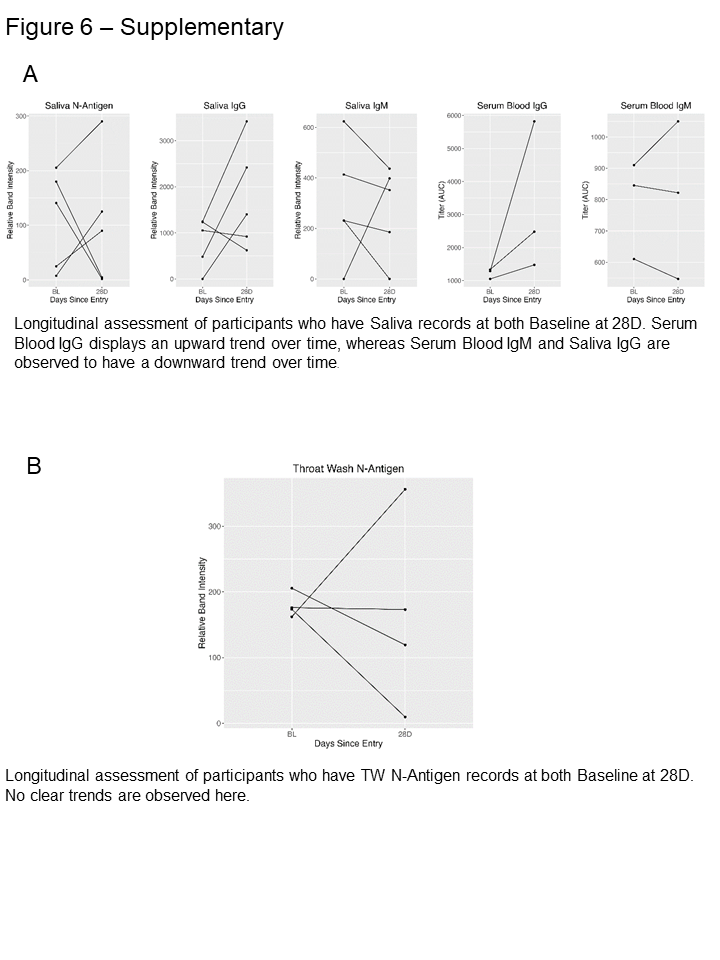

Supplement: Supplementary file 10 — Supplementary Information 10. [file 41598_2024_67504_MOESM10_ESM.tif]
